# The incretin–joint axis: weight-independent mechanisms and disease-modifying potential in chronic arthropathies

**DOI:** 10.1093/rap/rkag105

**Published:** 2026-08-18

**Authors:** Nefeli Stavroula Papastathopoulou, Simona Neri, Elisa Assirelli, Andrea D’Amuri, Salvatore Greco, Jacopo Ciaffi, Francesco Ursini

**Affiliations:** Medicine & Rheumatology Unit, IRCCS Istituto Ortopedico Rizzoli, Bologna, Italy; Medicine & Rheumatology Unit, IRCCS Istituto Ortopedico Rizzoli, Bologna, Italy; Medicine & Rheumatology Unit, IRCCS Istituto Ortopedico Rizzoli, Bologna, Italy; General Medicine Unit, ASST Mantova—Ospedale Carlo Poma, Mantova, Italy; Internal Medicine Unit, Ospedale del Delta, Lagosanto, Italy; Medicine & Rheumatology Unit, IRCCS Istituto Ortopedico Rizzoli, Bologna, Italy; Department of Biomedical and Neuromotor Sciences, Alma Mater Studiorum University of Bologna, Bologna, Italy; Medicine & Rheumatology Unit, IRCCS Istituto Ortopedico Rizzoli, Bologna, Italy; Department of Biomedical and Neuromotor Sciences, Alma Mater Studiorum University of Bologna, Bologna, Italy

**Keywords:** osteoarthritis, rheumatoid arthritis, psoriatic arthritis, incretin, GLP-1, GIP, semaglutide, tirzepatide

## Abstract

Chronic arthropathies are major drivers of pain and disability, and an immunometabolic template is reshaping how osteoarthritis (OA) and inflammatory arthritis are conceptualized, linking systemic metabolic stress to synovial inflammation, cartilage catabolism and disease progression. Incretin-based therapies (GLP-1RAs and dual GLP-1R/GIPR agonists) have revealed pleiotropic actions extending beyond weight loss and glycaemic control, supporting the concept of an ‘incretin–joint axis’ with disease-modifying potential. Joint tissues appear capable of sensing incretin signalling, supporting a direct, receptor-mediated action within the joint. Across articular cell types, GLP-1RA exposure engages conserved stress-response pathways, reducing inflammation and catabolism in chondrocytes and modulating synovial fibroblast and macrophage activity in inflammatory arthritis. Early clinical data indicate improvements in OA symptoms with GLP-1R agonism; however, mechanistic attribution remains intertwined with weight reduction. Definitive translation will require receptor-centric mapping in human joint microenvironments and interventional designs disentangling direct joint actions from weight-mediated benefit.

Key messages

- Incretin-based therapies may be able to exert weight-independent, direct effects on joint cells.
- Incretin receptor signalling has demonstrated anti-inflammatory and anti-catabolic effects in experimental joint tissues.
- Clinical studies must determine whether these effects translate into meaningful rheumatological benefit.

## Introduction

Chronic arthropathies are among the most prevalent causes of long-term pain and disability worldwide, yet—despite major therapeutic advances in inflammatory arthritis—durable, mechanism-aligned disease modification remains elusive for some patient groups. Osteoarthritis (OA) alone is projected to affect close to one billion people by 2050, driven by population ageing and the expanding prevalence of obesity, with an associated rise in disability and health-care demand [1]. Rheumatoid arthritis (RA), while less prevalent, continues to exert a substantial global burden, with estimates of 17.6 million affected individuals in 2020 and increasing prevalence over recent decades [2]. Psoriatic arthritis (PsA) similarly contributes disproportionate morbidity, amplified by cardiometabolic comorbidity and obesity, which are highly prevalent in this population [3].

In parallel with increasingly effective immunomodulatory strategies in RA and PsA, a complementary paradigm has gained traction: arthritis is not only ‘immune’ or ‘mechanical’, but also deeply shaped by immunometabolic stress [4]. In OA, in particular, convergent epidemiological, clinical and experimental data support a metabolically modulated, low-grade inflammatory component that influences pain, tissue remodelling and progression, extending beyond joint loading alone [5]. This reframes arthritis within systemic immunometabolic stress networks that can modulate synovial and cartilage biology.

Glucagon-like peptide-1 receptor agonists (GLP-1RAs), and more recently dual GLP-1/glucose-dependent insulinotropic polypeptide (GIP) receptor agonists, have transformed the treatment landscape for type 2 diabetes and obesity and have revealed clinically meaningful pleiotropy extending beyond glycaemic control [6]. A particularly important ‘proof of relevance’ for rheumatology is the STEP-9 trial [7], in which once-weekly semaglutide (2.4 mg) over 68 weeks improved knee OA pain and physical function in individuals with obesity, alongside substantial weight loss—clinically important, yet mechanistically inseparable by design. In parallel, trials such as STOP KNEE-OA (NCT06191848) are explicitly testing whether long-term incretin-based therapy (tirzepatide) can alter harder outcomes, including progression to knee replacement, in patients with obesity and knee OA [8, 9].

What has changed in the last few years is the mechanistic plausibility that incretin biology may act within arthritic tissues, not merely around them. Across diverse experimental systems, GLP-1–based medicines have been shown to modulate inflammation through a combination of systemic metabolic improvements and direct actions on immune and non-immune cells—the cellular ecology that sustains synovitis, oxidative stress and tissue-destructive signalling in arthritis. Yet the evidence remains scattered: cartilage biology is often discussed separately from synovium; innate immune effects are siloed from stromal and bone remodelling; and OA, RA and PsA literatures rarely cross-pollinate despite shared immunometabolic circuitry.

In this review, we weave these strands into a joint-centred framework, synthesizing evidence for weight-independent, receptor-mediated actions across joint-resident and immune cell compartments, and critically appraising emerging translational and clinical data to distinguish direct tissue-level biology from the substantial—yet clinically meaningful—effects of weight loss.

## Basic pharmacology of incretin analogues

Physiologically, the incretin effect denotes the amplification of insulin secretion elicited by oral glucose or mixed meals compared with isoglycaemic intravenous glucose challenge; a phenomenon mediated largely by the gut-derived incretin hormones GLP-1 and GIP [10]. GLP-1 is secreted post-prandially by enteroendocrine L cells, with circulating concentrations rising several-fold after nutrient ingestion [11]. It supports glucose homeostasis and satiety by potentiating glucose-dependent insulin secretion, suppressing glucagon during hyperglycaemia and slowing gastric emptying, thereby attenuating postprandial glycaemic excursions and reducing food intake [11].

GIP is secreted by enteroendocrine K cells—predominantly in the proximal small intestine—in response to nutrient ingestion, and it contributes substantially to postprandial insulin secretion [12–14].

GLP-1R is most abundantly expressed in pancreatic islets, the central nervous system and the gastrointestinal tract, but is also present in multiple peripheral tissues [15]. In β cells, GLP-1R couples predominantly to Gαs, increasing intracellular cAMP and engaging downstream effectors such as protein kinase A (PKA) and Epac2, thus potentiating glucose-stimulated insulin secretion [16, 17].

The GIP receptor (GIPR) is likewise a class B1 GPCR that generally signals through Gαs/cAMP; however, its net impact on islet hormone output is shaped by intra-islet paracrine interaction and signalling bias [14, 18].

Consistent with a pleiotropic biology, GLP-1R and GIPR are expressed across multiple organs and cell types beyond the pancreas, providing a mechanistic basis for non-glycaemic effects [19–21]. In the central nervous system, incretin receptor signalling contributes to appetite regulation and satiety [19, 20]. Preclinical evidence indicates that GIPR signalling in immune/myeloid compartments can restrain macrophage-driven inflammatory programs, whereas GLP-1R expressed on gut intraepithelial lymphocytes has been implicated in modulation of local intestinal inflammatory tone [19, 20]. Clinically, GLP-1R agonists have consistently reduced major adverse cardiovascular events in randomized cardiovascular outcome trials and meta-analyses [22], with emerging evidence suggesting that cardiovascular benefits may also extend to patients with autoimmune rheumatic diseases [23].

Native GLP-1 has a very short half-life due to rapid degradation by dipeptidyl peptidase IV (DPP-4) and renal clearance, limiting its therapeutic usability [11, 15, 24]. Therapeutic GLP-1 analogues are therefore engineered to resist DPP-4 degradation and prolong systemic exposure [11, 15, 24]. In parallel, GIPR has emerged as a therapeutic target, with dual GLP-1/GIP receptor agonism demonstrating superior efficacy in weight reduction and glycaemic control compared with GLP-1R agonism alone [25, 26]. Reflecting the growing interest in extrapancreatic biology, tirzepatide (a dual GLP-1/GIP receptor agonist) has received U.S. FDA approval for the treatment of moderate-to-severe obstructive sleep apnoea in adults with obesity and is being evaluated for additional cardiometabolic and inflammatory indications [27–29].

## Tissue distribution of incretin receptors in the joint

Available evidence concerning tissue distribution of incretin receptors in the joint is summarized in Table 1.

**Table 1:** Evidence for GLP-1R and GIPR expression and effects in joint-related cell types.

Cell type	Receptor expression	Direct effects of incretin signalling	Level of evidence	Key references
Chondrocytes	GLP-1R (murine and human)	↓ inflammation; ↓ catabolism; ↓ oxidative stress; ↑ survival	*In vivo, in vitro*	[31–33, 75, 91]
Synovial fibroblasts	GLP-1R (human synovial membrane and equine synoviocytes)	↓ inflammation; ↓ MMPs; ↓ oxidative stress	*In vitro*	[31, 34, 76–78]
Macrophages	GLP-1R (murine and human); GIPR (human)	M2 (re)polarization; ↓ pro-inflammatory signalling	*In vitro*	[31, 35, 36, 45–48, 81]
Osteoblasts	Maturation-dependent pattern of GLP-1R (human preosteoblasts); GIPR detected (human)	↑ Survival; ↑ osteogenesis; ↑ bone mass; ↓ bone resorption; ↓ apoptosis	*In vivo, in vitro, ex vivo*, clinical studies	[49, 51, 52, 54, 55, 60, 84, 85]
Bone marrow stromal cells	GLP-1R (murine and human)	↑ survival; ↑ osteogenesis	*In vitro*	[61]
Osteoclasts	GIPR (human); GLP-1R inconsistent or undetected (murine and human)	GLP-1 indirect effects	*In vivo, in vitro*	[55–57, 60]
Tenocytes	GLP-1R (murine and human)	↓ inflammation; ↓ apoptosis; ↑ mitochondrial function and survival; ↑ tendonogenic differentiation	*In vivo, in vitro*	[64, 65]
Endothelial cells	GLP-1R and GIPR, but not bone/joint specific	↓ inflammation; ↓ adhesion molecules; ↓ ER stress; ↑ mitochondrial integrity	*In vivo, in vitro*	[19, 69, 71, 88, 89]

**Abbreviations:** GLP-1R, glucagon-like peptide-1 receptor; GIPR, glucose-dependent insulinotropic polypeptide receptor; MMPs, matrix metalloproteinases; ER, endoplasmic reticulum; M2, anti-inflammatory macrophage phenotype.

**Footnotes: **  *in vivo*, *in vitro*, and *ex vivo* refer to experiments performed in living organisms, isolated cells/tissues, or freshly isolated tissues, respectively. Arrows indicate direction of effect (↑ increase; ↓ decrease).

### Cartilage

GLP-1R expression has been detected in human and murine articular cartilage and in primary human chondrocytes [30, 31]. In a murine OA model, intra-articular GLP-1R blockade abolished the cartilage-protective effects associated with experimentally increased endogenous GLP-1, supporting a direct, local role for GLP-1R signalling in modulating cartilage degeneration [30].

Beyond presence, available evidence suggests disease-modulated receptor expression. GLP-1R expression appears reduced in osteoarthritic cartilage and declines with increasing OA severity in experimental models, suggesting that receptor downregulation may accompany disease progression [32, 33]. At present, direct evidence for GIPR expression or functional signalling in articular chondrocytes remains unavailable.

### Synovium

Compared with cartilage, evidence for GLP-1R expression in synovial tissue is more limited, but supports receptor presence in key synovial compartments [31, 34]. GLP-1R immunoreactivity has been detected in the vascular and intimal lining compartments of human osteoarthritic synovium and in synovial intimal cells in an equine model. However, since the intimal lining contains both macrophage-like and fibroblast-like synoviocytes (FLS), these data do not definitively localize receptor expression to FLS specifically. Direct evidence for GIPR expression in synovial cells is currently lacking.

### Immune compartments

A substantial body of experimental literature indicates that GLP-1R expression extends in generally modest to low amounts across innate and adaptive immune compartments, although its abundance and functional relevance vary considerably across immune cell populations [35, 36]. Evidence suggests that lymphoid cells—and particularly the intestinal intraepithelial lymphocytes—express the GLP-1 receptor more than the myeloid lineage [37, 38]. Despite RT-PCR identification of GLP-1R transcripts in human monocytes and monocyte-derived macrophages [35, 36], research has revealed that GLP-1R agonism attenuates myeloid cell-driven inflammation indirectly, by acting on central neuronal GLP-1 receptors [37, 39]. Nevertheless, complementary protein-level evidence has been provided in murine macrophage systems, where immunohistochemical and immunofluorescence analyses detected GLP-1R staining in RAW 264.7 macrophages, at both membrane and intracellular locations [31]. These findings should be interpreted with caution because low receptor expression and methodological limitations in receptor transcript or antibody-based detection complicate cell-type specific localization and functional understanding.

Low GLP-1R expression has also been documented across adaptive immune lineages [40–44]. A multi-cohort study reported GLP-1R on circulating murine and human T and B cells, and on human invariant natural killer T (iNKT) cells, consistent with broad lymphocyte distribution [40–42]. Additional data indicate GLP-1R transcripts in immature lymphocyte populations and in CD4^+^CD25^+^ subsets, suggesting potential relevance to lymphocyte maturation and regulatory pathways [40–42].

Parallel evidence supports the presence of GIPR in myeloid cells, with GIPR mRNA reported in peripheral blood mononuclear cells, monocytes and monocyte-derived macrophages [45–48]. Importantly, murine functional studies indicate that macrophages are GIP-responsive, as genetic or pharmacological disruption of GIPR signalling in myeloid lineages alters macrophage-driven inflammatory phenotypes [45, 46]. Collectively, these findings suggest that GLP-1R/GIPR expression is not confined to classical metabolic tissues but extends moderately to immune populations central to inflammatory and autoimmune processes relevant to rheumatic disease, which may allow them to influence pathological immune pathways. Evidence supporting functional myeloid GIPR signalling is currently stronger than supporting direct myeloid GLP-1R signalling, since the functional relevance of GLP-1R agonists seems more indirect and mediated through neuronal pathways.

### Bone

Evidence for GLP-1R expression in the osteoblast lineage is heterogeneous but generally suggests greater expression in stromal, progenitor and immature osteoblast populations than in mature osteoblasts [49–52]. GLP-1R expression has also been observed during osteogenic differentiation of human adipose-derived stem cells, further implicating GLP-1 signalling in early osteoblastic commitment [53].

In contrast to the variable GLP-1R signal, evidence for GIPR expression in the osteoblast lineage is comparatively robust at both transcript and protein levels [52, 54, 55]. Together, these data position osteoblast lineage cells as established GIPR-expressing targets with clear structural plausibility for direct GIP signalling.

Evidence for direct GLP-1R expression in osteoclasts is inconsistent, whereas GIPR expression and functional responsiveness are more consistently supported [56, 57]. Nevertheless, GLP-1R agonists can reduce inflammatory bone resorption *in vivo*, largely through indirect mechanisms, including immune modulation and suppression of pro-osteoclastogenic cytokines such as TNF-α [58, 59].

By contrast, multiple lines of evidence support GIPR expression in osteoclast lineage cells, establishing osteoclasts as GIP-responsive targets [55, 60]. This receptor asymmetry between GLP-1R and GIPR offers a mechanistic basis for potentially distinct—and possibly complementary—effects of single versus dual incretin receptor agonism on bone resorption.

Furthermore, GLP-1R expression has been demonstrated in human bone marrow stromal cells (BMSCs) and in rat BMSCs [61], alongside reports again suggesting absent or low detectability in primary mature osteoblasts [50]. Given the role of stromal cells and early osteoblastic precursors in subchondral remodelling, osteophyte formation and erosive bone changes in OA and RA, GLP-1R signalling in these populations may be directly relevant to joint pathology, potentially independently of systemic metabolic effects [62]. To date, direct evidence for GIPR expression on bone marrow mesenchymal stem/stromal cells remains lacking; available studies instead implicate GIPR-expressing niche cells—particularly osteoblast-lineage populations—in regulating hematopoietic stem and progenitor maintenance [63].

### Tendon

GLP-1R expression has been directly demonstrated in tenocytes [64], and additional studies suggest GLP-1R signalling can modulate tenocyte function, although receptor localization has not been uniformly assessed across reports [65]. In contrast, incretin receptor mapping in enthesis-resident cells remains essentially unexplored, and direct evidence for GLP-1R/GIPR localization in enthesis fibrocartilage cells is currently lacking. Likewise, dedicated studies assessing GIPR expression in tenocytes have not been reported.

### Blood vessels

GLP-1R and GIPR signalling has been extensively characterized in vascular endothelial biology in the context of atherosclerosis and cardiovascular disease [66–68], and within broader cardiometabolic clinical frameworks [19, 69–71]. However, direct incretin-receptor localization specifically within bone or synovial microvascular endothelium has not been systematically examined.

## Effect of incretin analogues on cells of the synovial joint

**Figure 1:**
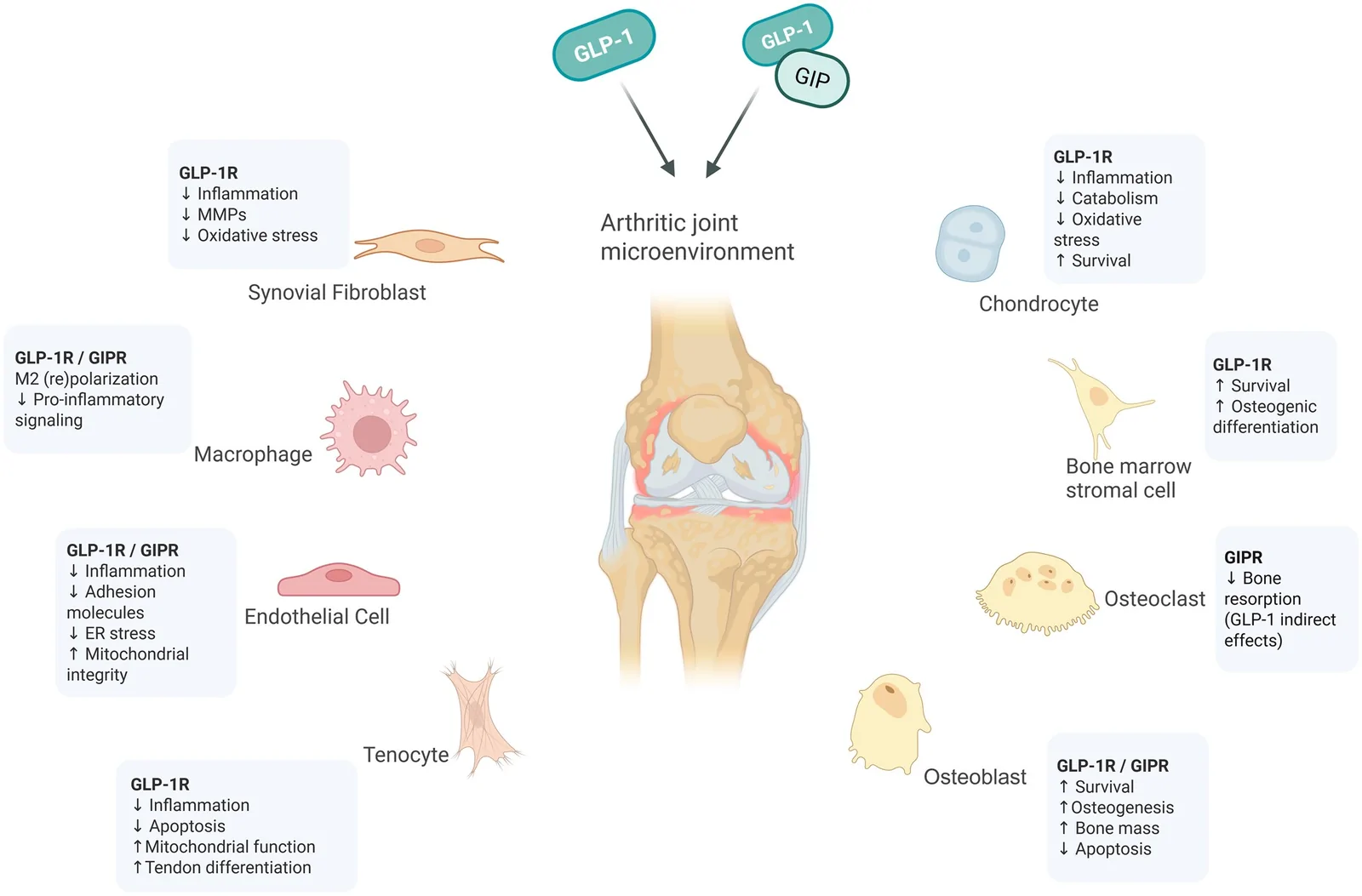
Reported incretin receptor effects in the osteoarthritic joint. Schematic representation of proposed/reported receptor-mediated, weight-independent effects of incretin-based therapies on cellular components of the osteoarthritic joint microenvironment. Activation of GLP-1R and GIPR modulates inflammatory signalling, oxidative stress, apoptosis, survival, differentiation and bone remodelling pathways in chondrocytes, synovial fibroblasts, macrophages, endothelial cells, BMSCs, osteoblasts, osteoclasts and tenocytes. Arrows indicate the direction of effect (↑ increase; ↓ decrease). MMPs, matrix metalloproteinases; ER stress, endoplasmic reticulum stress. GLP-1, glucagon-like peptide-1; GIP, glucose-dependent insulinotropic polypeptide

The reported direct effects of incretin analogue signalling across the cellular components of the synovial joint are summarized in Table 1 and illustrated schematically in Fig. 1.

### Chondrocytes

In human SW1353 chondrocytes exposed to advanced glycation end products (AGEs), dulaglutide reduced the expression of key pro-inflammatory mediators (IL-6, IL-8, MCP-1, COX-2 and PGE2) and cartilage-degrading enzymes (MMP-3, MMP-13, ADAMTS-4 and ADAMTS-5) [72]. These effects were accompanied by attenuation of AGEs-induced oxidative stress and preservation of extracellular matrix components (type II collagen and aggrecan), and were mechanistically linked to inhibition of NF-κB signalling [72]. Notably, AGEs exposure reduced GLP-1R expression in this model, suggesting that degenerative stress may blunt endogenous incretin responsiveness [72].

Complementary *in vitro* studies further indicate that liraglutide counteracts cytokine-driven chondrocyte activation in a dose-dependent manner [31, 73]. In murine chondrocytes, liraglutide suppressed IL-1β–induced inflammatory outputs (including IL-6, prostaglandin E2 and nitric oxide) and downregulated ADAMTS-4/ADAMTS-5 at both mRNA and protein levels [31, 73]. In human chondrocytes, liraglutide attenuated TNF-α–induced upregulation of IL-6 and MCP-1 and reduced expression of MMP-3 and MMP-13, consistent with a coupled anti-inflammatory and anti-catabolic activity [31, 73]. Mechanistically, these effects aligned with marked inhibition of NF-κB transcriptional activity and were accompanied by reduced oxidative stress and NOX4 expression, supporting a receptor-dependent antioxidant component [31, 73].

More recent work suggests that GLP-1R agonism may also directly preserve chondrocyte viability under glycoxidative stress. In primary chondrocytes exposed to AGEs, liraglutide suppressed RAGE signalling, limiting inflammation, apoptosis and matrix catabolism; it reduced MMP-1, MMP-3, MMP-13 and ADAMTS-4/5, while restoring type II collagen and aggrecan [74]. Importantly, these effects were reversed by GLP-1R blockade, reinforcing receptor specificity [74].

Across additional experimental settings, GLP-1R activation consistently produced pro-survival and anti-apoptotic effects in chondrocytes [32, 75]. In primary human chondrocytes challenged with AGEs, GLP-1R activation restored mitochondrial function, reduced apoptosis and preserved extracellular matrix components [32, 75]. In IL-1β-treated chondrocytes, GLP-1R agonism reduced apoptosis markers (including cleaved caspase-3 and Bax) and increased Bcl-2, with mechanistic experiments indicating dependence on PI3K/Akt signalling and association with attenuation of endoplasmic reticulum stress and inhibition of NF-κB activation [32, 75]. In the same experimental framework, liraglutide ameliorated cartilage degeneration in a rat OA model [32, 75].

*In vivo* data support these cellular observations. In rat OA models, cartilage GLP-1R expression declined with increasing disease severity in parallel with reduced PKA/CREB signalling and increased IL-1β, IL-6 and TNF-α [33]. Liraglutide treatment restored GLP-1R expression and PKA/CREB activation and reduced inflammatory cytokine levels in cartilage tissue, consistent with a local, weight-independent protective effect on articular cartilage [33].

To date, no studies have specifically examined the effects of dual GLP-1/GIP receptor agonists (e.g. tirzepatide) on articular chondrocytes.

Taken together, these studies indicate that GLP-1R agonists directly modulate inflammatory, oxidative and catabolic pathways in chondrocytes and promote cell survival, resulting in improved matrix preservation and cartilage homeostasis under osteoarthritic conditions.

### Fibroblast-like synoviocytes

In primary human RA FLS, exenatide attenuated TNF-α–induced oxidative stress, reduced release of pro-inflammatory cytokines, suppressed matrix metalloproteinase production, and inhibited activation of p38 and NF-κB signalling, consistent with a direct GLP-1R–mediated anti-inflammatory effect [76]. These findings were corroborated in a subsequent study in which dulaglutide restored mitochondrial membrane potential, reduced NOX4-driven oxidative stress, suppressed IL-1β, IL-6, MCP-1 and HMGB1, and downregulated MMP-3 and MMP-13; mechanistically, these effects were mediated through inhibition of JNK and NF-κB signalling [77].

Similarly, lixisenatide markedly attenuated inflammatory responses and mitochondrial dysfunction in human RA FLS, reducing IL-1β–induced ROS generation and cytotoxic 4-hydroxynonenal production [78]. Consistent with the broader pattern observed across GLP-1R agonists, lixisenatide inhibited key pro-inflammatory signalling programs (including JNK/AP-1 and NF-κB) and reduced inflammatory mediators and metalloproteinases central to synovial cytokine amplification and tissue-destructive pathways in RA [78].

To date, no studies have directly investigated the effects of dual GLP-1/GIP receptor agonists (e.g. tirzepatide) on fibroblast-like synoviocytes.

### Macrophages

Macrophages represent a key joint-resident and infiltrating population [79] through which incretin signalling may influence inflammatory tone and resolution pathways.

Early *in vitro* evidence demonstrated that GLP-1/GLP-1R signalling is linked to STAT3 activation and macrophage polarization, favouring an anti-inflammatory M2 phenotype [35]. This is conceptually aligned with established macrophage functional heterogeneity, in which classically activated M1 macrophages amplify inflammation and tissue damage, whereas alternatively activated M2 macrophages support resolution and repair [80]. In a rat model of spinal cord injury, exenatide shifted macrophage profiles toward an M2 phenotype, with increased M2 markers and anti-inflammatory interleukins alongside reduced M1 markers and pro-inflammatory cytokines [81]. *In vitro*, liraglutide similarly promoted repolarization from M1 toward M2, although the extent to which these effects reflect direct macrophage GLP-1R signalling remains uncertain [31].

Beyond GLP-1R, functional evidence indicates that GIP can also directly modulate macrophage inflammatory activity via GIPR-dependent mechanisms [45, 48]. In murine models of metabolic disease and atherosclerosis, GIP administration attenuated macrophage-driven inflammation, reduced foam cell formation and suppressed atherosclerotic lesion development; these effects were abrogated by pharmacological GIPR antagonism [45, 48]. Complementary genetic evidence showed that myeloid cell-specific deletion of GIPR exacerbated inflammatory and metabolic dysfunction through dysregulation of the S100A8/A9 alarmin axis, implicating a GIPR-S100A8/A9 pathway in restraining macrophage-driven inflammation [45, 48]. Although these studies are not joint-specific, they provide a mechanistic framework supporting macrophages as functionally relevant targets of direct GIP signalling, with potential implications for inflammatory pathways pertinent to rheumatic disease.

### Osteoblasts and osteoclasts

Experimental evidence indicates that GLP-1R agonists exert biologically meaningful effects on bone-related cell populations, with signalling that appears to be concentrated in stromal/progenitor compartments rather than terminally differentiated osteoblasts [50]. In an *in vivo* rat study, exendin-4 improved bone mass, formation and quality despite absent detectable GLP-1R expression on mature osteoblasts; bone marrow stromal cells were identified as the primary GLP-1R-expressing population mediating osteogenic differentiation via PKA/β-catenin and PKA/PI3K/Akt pathways [50]. More broadly, GLP-1R agonists have been reported to promote osteogenic differentiation and inhibit bone resorption [82] through pathways central to remodelling and often implicated in joint pathology, including PI3K/Akt, MAPK, Wnt/β-catenin, and modulation of the OPG/RANKL ratio [82]. Supporting these concepts, a 2025 study reported that semaglutide improved bone microarchitecture in ovariectomized rats, increased β-catenin expression, and suppressed RANKL activation, consistent with enhanced osteoblast activity and reduced osteoclast-mediated resorption [83]. Together, these data suggest a plausible route by which GLP-1R agonism could influence subchondral bone remodelling, a process increasingly recognized as relevant to cartilage degeneration and disease progression in OA and inflammatory arthritis.

In parallel, the evidence base for GIP in skeletal biology is comparatively extensive and supports coordinated actions on bone-forming and bone-resorbing cells [54, 84]. In osteoblast-lineage cells, GIP induces dose-dependent increases in intracellular cAMP and calcium signalling and upregulates collagen type I expression and alkaline phosphatase activity, consistent with anabolic osteoblastic responses [54, 84]. *In vivo*, disruption of GIPR signalling is associated with reduced bone formation, increased osteoclast number and a high-turnover osteoporotic phenotype, whereas GIP exposure reduces osteoblast apoptosis and links postprandial calcium availability to skeletal calcium deposition [54, 84]. At the level of bone resorption, GIP directly suppresses osteoclast differentiation and activity [55, 60]. *In vitro* and *ex vivo* studies demonstrate inhibition of osteoclastogenesis, delayed resorption and increased osteoclast apoptosis through modulation of intracellular pathways including cAMP, Akt, p38, Src and NF-κB, with downstream impairment of NFATc1 nuclear translocation; importantly, these actions are receptor-dependent and are abolished by GIPR antagonism [55, 60]. Clinically, exogenous GIP administration in healthy humans reduces bone resorption and increases bone formation markers, supporting a physiological role in postprandial bone turnover, although these systemic markers do not provide cell-specific mechanistic resolution in humans [85].

The development of *maridebart cafraglutide*, an agent combining GLP-1R agonism with GIPR antagonism, further complicates this mechanistic framework [86]. Although this approach produces substantial weight loss [87], GIPR antagonism could theoretically mitigate some of the potentially beneficial direct effects of GIPR signalling on osteoblasts, osteoclasts and myeloid cells; consequently, its net effects on joint inflammation and bone remodelling remain uncertain and warrant further investigation.

### Tenocytes

Emerging evidence suggests incretin mimetics can directly modulate tendon cell survival and reparative programs [64, 65]. In tenocytes, liraglutide attenuated IL-1β–induced inflammatory injury and apoptosis by upregulating GLP-1R expression and engaging AMPK/SIRT1 signalling, with preservation of mitochondrial function, mitigation of endoplasmic reticulum stress, and suppression of apoptotic pathways [64, 65]. *In vivo*, GLP-1R activation was associated with improved tendon outcomes in a rat rotator cuff injury model, supporting a receptor-dependent contribution to tissue repair [64, 65]. Complementary work indicates that exendin-4 promotes tendonogenic differentiation of human mesenchymal stem cells, increasing expression of tendon-associated transcription factors (including Scx and Mkx) and extracellular matrix components such as type I collagen, decorin and tenascin-C [64, 65].

### Endothelial cells

GLP-1R agonists also exert direct anti-inflammatory and cytoprotective effects on vascular endothelial cells, mechanisms that may be relevant to inflammatory joint microenvironments by influencing endothelial activation, immune cell trafficking and amplification loops [31, 88, 89]. In human endothelial models, liraglutide reduced activation induced by TNF-α, lipopolysaccharide, or hyperglycaemic stress, with decreases in adhesion molecule expression (e.g. VCAM-1, E-selectin), reduced monocyte adhesion, and attenuation of endoplasmic reticulum stress responses [31, 88, 89]. These effects were associated with intracellular Ca²^+^ signalling and activation of CaMKKβ-AMPK, leading to downstream engagement of eNOS and CREB and suppression of endothelial inflammatory signalling [31, 88, 89]. In parallel, liraglutide preserved mitochondrial integrity and reduced apoptosis under hyperglycaemic stress by modulating mitochondrial dynamics and ER stress–associated pro-apoptotic pathways [31, 88, 89].

## Beyond weight loss: disease-specific evidence for incretin analogue actions

### Osteoarthritis

Accumulating experimental and early clinical evidence supports a potential disease-modifying role for GLP-1R signalling in OA [90]. In preclinical models, GLP-1R expression appears to decline over the course of OA development [33]. In monosodium iodoacetate (MIA)-induced OA rat knees, progressive receptor downregulation is accompanied by suppression of PKA/CREB signalling and increased expression of inflammatory mediators [33]. Restoration of GLP-1R signalling with liraglutide reactivated PKA/CREB pathways and attenuated inflammatory protein expression, consistent with the hypothesis that loss of GLP-1R signalling may contribute to OA pathogenesis [33].

The causal relevance of local receptor engagement is further supported by *in vivo* intervention studies. Intra-articular blockade of GLP-1R completely abolished the cartilage-protective effects observed when endogenous GLP-1 was experimentally increased in OA mice, and local administration of liraglutide mitigated cartilage degeneration, providing direct evidence for weight-independent, joint-local GLP-1R–mediated protection [30, 31]. Consistent with this framework, lixisenatide administered at clinically relevant doses demonstrated protective effects across multiple OA-relevant domains, including preservation of the articular extracellular matrix, mitigation of mitochondrial dysfunction and oxidative stress, suppression of proinflammatory cytokine expression, and inhibition of NF-κB activation [75]. Across these models, pharmacological GLP-1R activation converges on a coherent mechanistic signature—restoration of anabolic signalling (including PKA/CREB), dampening of inflammatory pathways (including NF-κB), and preservation of cartilage structural integrity—supporting an effect profile that extends beyond symptomatic relief alone [33, 75].

Translational observations are broadly consistent with these experimental findings. GLP-1R has been detected in human osteoarthritic cartilage and synovial tissue and intra-articular liraglutide reduced inflammatory mediator production and cartilage breakdown in murine OA models [92]. Clinical evidence supporting GLP-1R agonists in OA is emerging but remains largely concentrated in obesity-associated OA, where mechanical unloading and metabolic improvements are intrinsically intertwined [7, 9]. In the STEP 9 randomized, double-blind, placebo-controlled trial, once-weekly semaglutide (2.4 mg) administered for 68 weeks to individuals with obesity and moderate knee OA produced significantly greater reductions in body weight and knee pain (WOMAC score), with improved physical function, compared with placebo [7]. While these findings establish meaningful symptomatic benefit, they do not allow definitive attribution to direct joint-specific mechanisms independent of weight loss. In the observational Shanghai Osteoarthritis Cohort, GLP-1RA exposure in patients with knee OA and type 2 diabetes was associated with fewer knee surgeries, improved pain and slower cartilage loss; mediation analysis estimated that weight reduction accounted for 32.1% of the association with reduced knee surgery, suggesting that weight loss explained part—but not necessarily all—of the observed benefit [93]. In parallel, trials like STOP KNEE-OA are evaluating whether long-term incretin-based therapy (specifically tirzepatide) can reduce the need for knee replacement surgery in obese patients with knee OA, reflecting growing clinical interest in potential disease modification [9]. However, because current clinical studies are either weight-centric by design or still recruiting, the human evidence base remains insufficient to clearly separate weight-dependent mechanical unloading from the direct, weight-independent biological effects on cartilage, synovium, and subchondral bone suggested by preclinical models [7, 9].

### Rheumatoid arthritis

Rheumatoid arthritis is characterized by chronic synovial inflammation driven in large part by activated FLS, which acquire an aggressive phenotype and sustain cytokine production, oxidative stress and cartilage and bone destruction [94]. Experimental evidence suggests that GLP-1R agonists can directly modulate several of these pathogenic mechanisms in RA-relevant cell populations [78].

Multiple *in vitro* studies in primary human RA FLS have shown that GLP-1R agonism attenuates inflammatory and catabolic programs central to synovial pathology [76, 77]. Treatment with exenatide, dulaglutide or lixisenatide reduced TNF-α– or IL-1β–induced oxidative stress and mitochondrial dysfunction and suppressed the production of proinflammatory mediators (including IL-1β, IL-6, IL-8, MCP-1 and TNF-α) [76–78]. In parallel, GLP-1R activation reduced expression of matrix-degrading enzymes such as MMP-1, MMP-3 and MMP-13, supporting a direct inhibitory effect on pathways implicated in tissue destruction [76, 77]. Mechanistically, these protective actions were linked to inhibition of major inflammatory signalling cascades relevant to RA, including JNK/AP-1, p38 MAPK and NF-κB pathways [76, 78]. Together, these data suggest that GLP-1R agonists may interfere with core effector programs of RA synovitis, with a profile more consistent with disease-relevant pathway modulation than with purely symptomatic effects [78].

Genetic epidemiology further supports biological plausibility: population-level data indicate that higher GLP-1R gene expression is associated with lower odds of developing RA, although this association does not establish causality [91]. A recent population-based study, however, found no association between GLP-1RA exposure and reduced incidence of autoimmune rheumatic diseases, including RA, thereby tempering—but not disproving—the genetic signal [95]. In a small observational cohort of 30 patients with obesity, type 2 diabetes mellitus, and active RA, GLP-1R agonist exposure over 6 months was associated with improvements in morning stiffness, joint swelling, and finger pain, alongside reductions in CRP and erythrocyte sedimentation rate; the absence of a control group and concomitant ∼10% weight loss preclude separation of weight-dependent effects from direct immunomodulatory actions [96]. Overall, while cellular data support a direct anti-inflammatory and anti-catabolic role of GLP-1R agonists in RA-relevant effector cells, the field is limited by the scarcity of *in vivo* RA model evidence and the absence of controlled interventional trials in RA, underscoring the need for rigorous clinical testing [96].

### Psoriatic arthritis

Despite the well-established association between psoriatic disease, obesity and metabolic dysregulation, mechanistic evidence directly addressing weight-independent effects of GLP-1R agonism in PsA remains extremely limited [40, 97–100]. To date, preclinical studies specifically interrogating GLP-1R signalling in PsA-relevant joint tissues or immune compartments have not been reported.

Clinical data are preliminary. In a small, uncontrolled study in patients with PsA and obesity, liraglutide treatment was associated with improvements in oxidative stress markers (malondialdehyde), reductions in inflammatory parameters and improvements in disease activity indices including PASI and DLQI [101]; however, the lack of a control group and the limited sample size constrain interpretation and preclude causal inference beyond hypothesis generation. More recently, the TOGETHER-PsA randomized controlled trial showed that, among patients with active PsA and overweight with at least one weight-related comorbidity or obesity, concomitant treatment with tirzepatide and ixekizumab resulted in greater improvements in PsA disease activity, physical function, body weight, and quality of life than ixekizumab alone, with no new safety signals [102]. This trial, however, does not establish whether the rheumatological benefits were mediated by weight reduction, direct incretin-receptor signalling, an interaction with ixekizumab or a combination of these mechanisms. Overall, while early clinical observations and expert syntheses suggest plausibility—particularly in obesity-associated PsA—robust experimental studies and controlled clinical trials are required to determine whether GLP-1R agonism confers direct, disease-modifying effects on joint inflammation and structural outcomes in PsA [22, 101, 103].

## Conclusion and future perspectives

Accumulating preclinical and clinical evidence supports the plausibility that GLP-1RA and dual incretin agonists may elicit direct, weight-independent effects on joint-resident cells and immune pathways relevant to OA and RA, with only preliminary signals in PsA [104]. Across joint compartments, GLP-1R agonism appears to converge on anti-inflammatory, antioxidant, pro-survival and anti-catabolic pathways, although clinically meaningful structural or functional benefits in humans have yet to be demonstrated.

Favourable effects on osteoblasts, osteoclasts or skeletal muscle pathways in experimental models do not necessarily predict the net effects observed during clinical treatment, because GLP-1RA-associated weight loss may be accompanied by reductions in bone mineral density and absolute lean mass [105]. These trade-offs may be especially relevant in rheumatic populations, in whom osteopenia and sarcopenia are already prevalent. Future trials should therefore evaluate bone density, fractures, lean mass, muscle strength and physical function alongside rheumatological outcomes and examine whether exercise and nutritional interventions can mitigate these risks, since existing evidence appears heterogeneous [106–108].

Weight loss remains a major and clinically valuable mediator of benefit in OA, PsA and RA [8, 109–111]. Although *in vitro* studies, intra-articular interventions, and receptor-blockade experiments support direct joint effects, clinical studies such as STEP 9 and TOGETHER-PsA cannot reliably separate these mechanisms from mechanical unloading and systemic metabolic improvement [7, 9, 102].

From a clinical perspective, precisely separating weight-dependent from weight-independent benefit may be less important for patients with OA and obesity, for whom weight loss and symptomatic improvement are both desirable outcomes. Mechanistic separation remains important for patients without obesity; direct joint effects must ultimately translate into meaningful advantages and an acceptable risk-benefit balance.

Evidence maturity differs by disease: OA has the strongest preclinical and clinical foundation [112, 113], RA has compelling cellular but limited interventional evidence [114, 115], and evidence in PsA remains preliminary despite the recent TOGETHER-PsA trial [102, 103].

The effects of incretin signalling on stromal cells and bone remodelling, including the potentially contrasting consequences of GIPR agonism and antagonism, remain important areas for investigation; the central thesis, however, suggests that incretin signalling may act on the joint as an integrated organ, not as a collection of isolated tissues.

Ultimately, rethinking incretin-based therapies for rheumatic disease warrants cautious investigation rather than an assumption of disease modification. The evidence remains dominated by *in vitro* and non-human studies, with potential interspecies differences in receptor distribution and persistent confounding by weight loss in clinical studies.

Future research should prioritize receptor mapping and cell-specific analyses in human joint models, followed by controlled trials incorporating structural outcomes, clinically meaningful endpoints, and biomarkers of target engagement. Such trials should distinguish direct joint effects from weight-mediated benefits and determine whether metabolic phenotypes identify patients most likely to benefit.

## Data Availability

No new data were generated or analysed in support of this research.
